# Is it time to develop neurocritical care education in Brazil? Analysis of the Brazilian national survey on education in neurocritical care

**DOI:** 10.1055/s-0046-1816040

**Published:** 2026-02-27

**Authors:** Thire Baggio Machado Marazzi, Millene Rodrigues Camilo, Thiago Oscar Goulart, Pedro Vitale Mendes, Pedro Kurtz, Gisele Sampaio Silva, Octávio Marques Pontes-Neto

**Affiliations:** 1 Universidade de São Paulo, Faculdade de Medicina de Ribeirão Preto, Departamento de Neurociências e Ciências do Comportamento, Ribeirão Preto SP, Brazil.; 2 University of Toronto, Temerty Faculty of Medicine, Department of Neurology, Toronto ON, Canada.; 3 Harvard School of Public Health, Department of Epidemiology, Boston MA, United States.; 4 Universidade de São Paulo, Faculdade de Medicina, Departamento de Medicina Interna, São Paulo SP, Brazil.; 5 Instituto D'Or de Pesquisa e Ensino, Rio de Janeiro RJ, Brazil.; 6 Instituto Estadual do Cérebro Paulo Niemeyer, Rio de Janeiro RJ, Brazil.; 7 Universidade Federal de São Paulo, Escola Paulista de Medicina, Departamento de Neurologia, São Paulo SP, Brazil.; 8 Hospital Israelita Albert Einstein, Centro de Ensino e Pesquisa, São Paulo SP, Brazil

**Keywords:** Critical Care, Critical Care Outcomes, Education, Medical

## Abstract

**Background:**

Neurocritical care (NCC) education involves integrating knowledge and skills into various complex areas. While the USA, Canada, and Europe regulate didactic core training for medical residencies and fellowships, the quantity and quality of these training programs in Brazil remain unclear.

**Objective:**

To assess how NCC training is currently integrated into neurology and intensive care residency programs in Brazil.

**Methods:**

A cross-sectional survey composed of 27 multiple-choice and short-answer questions was distributed by email to professionals registered in national congresses and medical organizations. Data regarding exposure to NCC training, duration, supervision, infrastructure, and self-perceived competencies were analyzed.

**Results:**

A total of 208 responses from 82 centers across 14 Brazilian states were included. Respondents were primarily neurologists (58.2%) and intensivists (28.2%). Only 50.5% reported receiving NCC training during residency, typically lasting more than 4 weeks. Training predominantly occurred in intensive care units with 5 to 20 beds, supervised by intensivists. However, exposure to specialized skills was limited: 23% to transcranial Doppler, 21% to electroencephalogram (EEG) interpretation, and 24% to multimodal neuromonitoring. Confidence in managing complex cases was suboptimal, with 56% reporting confidence in postcardiac arrest care, 47% in refractory intracranial hypertension, and 40% in spinal cord trauma.

**Conclusion:**

Neurocritical care education in Brazil is heterogeneous and remains at an early stage of development. Most residents receive limited exposure to advanced neurocritical skills, resulting in low confidence to independently manage highly complex conditions. Standardized, competency-based training programs are urgently needed to enhance professional preparedness and potentially improve patient outcomes in NCC.

## INTRODUCTION

It is estimated that neurocritical patients account for 20 to 25% of all critically ill individuals requiring intensive care worldwide. 1 Neurological diseases represent one of the greatest global health burdens, due to their potential to cause sustained disability, long-term dependence, and elevated healthcare costs. Improved outcomes in mortality, length of hospital stay (LOS), long-term functional status, and healthcare expenditures have been consistently reported in neurovascular and traumatic brain injury (TBI) patients managed in centers with structured training, standardized protocols, and dedicated neurocritical care (NCC) units. 1 2 3 4 5 6 7 8 9 10

In response to this growing clinical and economic burden, academic and medical institutions worldwide began to develop specialized structures for the care of neurocritical patients. Since the 1980s, there has been a progressive expansion of academic institutions committed to advancing education and research in this field. 11 12 As a result, NCC has emerged as a multidisciplinary area that draws on expertise from neurology, neurosurgery, anesthesiology, internal medicine, emergency medicine, and radiology. 12 It focuses on the integrated and comprehensive management of patients with life-threatening neurological conditions that require specialized knowledge and decision-making skills.

In high-income countries, particularly the United States and Europe, NCC training has evolved to include mandatory core curricula, structured fellowship programs, and formal board certification. By 2018, the United States had 68 NCC fellowship programs accredited by the United Council for Neurologic Subspecialties (UCNS), in addition to 22 accredited by the Committee on Advanced Subspecialty Training (CAST), totaling 1,374 board-certified neurointensivists. 2 13 14 To better assess the scope, needs, and impact of NCC training, a series of nationwide surveys has been conducted over the past 2 decades, exploring clinical competencies, procedural exposure, educational methodologies, and program implementation over time. 15 16 17 18

In Brazil, however, the field remains in its early stages of development. 19 Neurocritical care is not formally recognized as a distinct subspecialty, and its training is not a mandatory component of either neurology or intensive care residency programs. No national curricular framework exists, and there are no formal mechanisms for certification or program accreditation at present.

According to the Neurocritical Brazil Study Group, approximately 10% of intensive care unit (ICU) admissions in Brazil between 2010 and 2023 were due to neurological causes. 20 Patients requiring NCC presented higher illness severity, longer ICU stays, and a mortality rate 1.7 times higher than that of non-neurocritical patients (17.2% vs 10.1%). These data highlight the substantial disease burden and resource demands associated with neurocritical conditions in the country, underscoring the need for specialized training and structured systems of care.

The most recent Brazilian Medical Demography (2025) 21 further illustrates this imbalance. The country currently has 5,866 neurologists and 10,412 intensive care physicians, but only 78 professionals hold dual certification in both specialties. This mismatch between clinical demand and trained workforce reinforces the need for national initiatives aimed at organizing and expanding NCC education.

Recent postgraduate and fellowship-style programs, mostly developed in academic centers and supported by collaborative initiatives, represent important pioneering efforts to formalize training. However, their limited number and geographic concentration illustrate the need for coordinated national strategies to expand and standardize NCC education in Brazil. 19

To our knowledge, this is the first nationwide effort to systematically characterize NCC training and practice in Brazil. By identifying strengths, challenges, and areas for improvement, and by comparing these findings with international benchmarks, the present study seeks to inform future efforts toward the structured development of NCC in the country.

## METHODS

The present is a descriptive, cross-sectional study based on a nationwide digital survey. The survey consisted of 27 questions, including both multiple-choice and short-answer formats ( **Supplementary Material** – available at https://www.arquivosdeneuropsiquiatria.org/wp-content/uploads/2025/11/ANP-2025.0302-Supplementary-Material.docx ). It was adapted from validated instruments 15 16 17 18 22 previously applied in the United States to evaluate NCC training and practice. The first item of the questionnaire was a digital informed consent form, which was required for participation.

The initial version of the instrument was developed by neurologists with expertise in critical care and was reviewed by three senior neurointensivists to ensure content validity. It was pilot tested among 76 respondents during academic events related to neurology and critical care. The final version covered five thematic domains:

- sociodemographic and professional profile;
- exposure to NCC during residency;
- postgraduate training models and academic paths;
- perception of educational gaps; and
- self-reported autonomy in managing neurocritical conditions.

Responses were collected anonymously between September and December 2023 using a secure digital platform.

To estimate the target population and define the expected sample size, we considered data from the Brazilian Academy of Neurology (ABN) and national medical workforce reports. According to the Brazilian Academy of Neurology (ABN, from the Portuguese Academia Brasileira de Neurologia), there are currently 96 accredited neurology residency programs in the country, graduating approximately 300 new specialists each year. 23 Since critical care medicine became an officially recognized medical specialty in Brazil in 2002, an estimated 5,400 neurologists have completed residency training nationwide. 24 Assuming a 95% confidence level and a 5% margin of error, the minimum required sample size was calculated to be 359 participants. Although the number of valid responses fell below this threshold, the geographic diversity and participation from both public and private institutions support the descriptive intent of the study and provide relevant insights.

Descriptive analyses were performed using the IBM SPSS Statistics for Windows (IBM Corp.) software, version 23.0. Figures were created using Python (Python Software Foundation). Artificial intelligence (AI) tools were used exclusively to support textual clarity and figure layout; no AI tools were used for data analysis or interpretation. Ethical approval was obtained from the local institutional review board (CAAE: 62305822.2.0000.5440).

## RESULTS

### Participant characteristics

A total of 208 valid responses were analyzed, representing professionals from 82 institutions across 14 Brazilian states ( Table 1 ). The mean participant age was 39 years, and 42% identified as female. Most respondents had completed their residency in public institutions (77.9%) and practiced neurology (58%) or intensive care medicine (29%). The Southeast region, particularly the state of São Paulo, contributed more than half of the responses, reflecting the concentration of academic centers and NCC training opportunities in Brazil. Only one respondent completed both neurology and intensive care residencies.

**Table 1:** Sociodemographic, clinical, and institutional profile of respondents

Variable		Value
Female gender (%)		42
Mean age (years); range		39.3 ± 10.43; 25–80
Mean years since graduation; range		21.4 ± 10.3; 1–52
Medical specialty (%)	Neurology	58.2
	Intensive care	28.8
	Neurosurgery	4.8
	Other	7.7
	Anesthesiology	0.5
Rotations (%)	Mandatory rotation	45.7
	Rotation at same institution as residency	36.5
Year(s) of rotation evaluation (%)	First year	25.0
	Second year	31.2
	Third year	15.9
	Other	3.8
Supervisor specialty (%)	Intensivist	43.8
	Neurologist	20.7
	Neurointensivist	12.0
	Neurosurgeon	5.8
	Anesthesiologist	1.4
Rotation duration in weeks (%)	> 8	15.9
	4–8	15.4
	2–4	17.3
	2	2.8
Type of institution (%)	Public	78
Number of beds (%)	< 5	1.0
	5–10	27.9
	10–20	22.6
	> 20	9.6
Geographic region of Brazil (%)	Southeast	73.3
	South	7.8
	Northeast	9.5
	North	0.0
	Midwest	9.5

These findings illustrate that the survey captured a diverse, yet regionally unbalanced sample, emphasizing the predominance of professionals trained in more developed regions with better access to structured educational programs.

A summary of participant demographics, training characteristics, and institutional affiliations is presented in Table 1 .

### Exposure to neurocritical care during residency

Half of the respondents (50.5%) reported having received formal NCC training during residency, most often as a mandatory rotation. However, only one third completed this training within their home institutions, indicating reliance on external centers for structured exposure. The proportion of participants reporting NCC training decreased with time since graduation, suggesting gradual recent incorporation into residency curricula. Supervision was predominantly provided by intensivists, with less frequent participation of neurologists or dedicated neurointensivists. Only 9% of respondents had completed postgraduate or fellowship training specifically in NCC.

### Structure and delivery of training

Training experiences were reported from 82 institutions across 14 Brazilian states, with most programs located in the southern and southeastern regions (Supplementary Material). Over half of the respondents (52.3%) completed rotations lasting more than 4 weeks, and nearly ⅓ (30.8%) for longer than 8 weeks. Training was predominantly conducted in general ICUs with 5 to 20 beds (78.8%) and supervised mainly by intensivists (43.8%), followed by neurologists (20.7%), and neurointensivists (12%).

Educational strategies were largely traditional: almost all participants (94.7%) reported bedside case discussions, while formal lectures (45.7%) and journal clubs (43%) were less common. Case presentations were reported by 38% of respondents, and simulation-based activities by only 6.6%, indicating that hands-on training through simulation remains rare.

Taken together, these data show that NCC education in Brazil is still primarily delivered through bedside learning in mixed ICUs, with limited structured teaching, and scarce exposure to simulation or competency-based educational formats.

### Clinical pathologies and training in procedures and monitoring

Respondents reported the neurological conditions in which they received practical training. Subarachnoid hemorrhage (SAH) and acute ischemic stroke (AIS) were the most frequently cited (both 95%), followed by TBI (92%), status epilepticus (SE) (88%), and brain death (85%).

Despite the high frequency of stroke-related training, only 46% of respondents reported experience managing patients after mechanical thrombectomy (MT). Although 82% had managed patients after aneurysm clipping, only 68% had experience with post-embolization care.

Training in neuromuscular disorders was less common. Guillain-Barré syndrome and myasthenia gravis had exposure rates of 75% and 59% respectively.

Participants were asked to indicate the neurological pathologies in which they received some form of training during their NCC experience.

Subarachnoid hemorrhage and AIS were the most frequently reported, both with a 95% training rate. However, only 68% had experience managing the postoperative period following aneurysm embolization, and 82% had exposure to aneurysmal clipping. Likewise, while stroke management was widely reported, only 46% of respondents had been involved in post-MT care.

Other frequent training exposures included TBI (92%), SE (88%), and brain death (85%). Neuromuscular diseases, such as Guillain-Barré syndrome (GBS) and myasthenia gravis (MG), were less commonly represented (75% and 59%, respectively), likely to reflect their lower prevalence and the concentration of care in referral centers.

Unexpectedly, low training rates were observed for several pathologies that are highly prevalent in Brazil and are often initially managed in non-tertiary settings. These included postcardiac arrest care (73%), refractory intracranial hypertension (71%), acute spinal cord injuries (60%), and central nervous system infections such as meningitis and encephalitis (69%).

Regarding procedural training, basic intensive care procedures were relatively common: central venous catheter placement (83%), orotracheal intubation (79%), arterial line insertion (65%), and dialysis catheter placement (47%). Nevertheless, considering the complexity of NCC patients, the authors anticipated higher exposure rates, close to universal, for such procedures. The findings suggest that many participants had only theoretical exposure (e.g., through lectures or bedside discussions), rather than hands-on clinical training.

Training in neurological monitoring was limited. Only 24% of respondents reported experience with multimodal monitoring. Electroencephalogram interpretation was part of the training for 30%, with only 20% having hands-on experience analyzing EEG traces. While 65% of participants received training in interpreting intracranial pressure (ICP) values and managing elevated ICP, only 11% reported having performed the placement of an invasive monitoring catheter. Transcranial Doppler (TCD) training was reported by 23%, consistent with the overall low exposure to bedside ultrasonography (31%).

**Figure 1:**
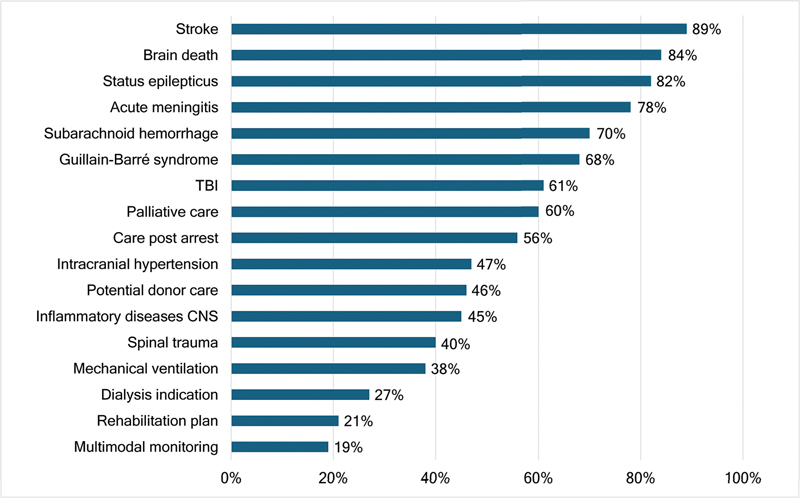
Abbreviation: TBI, traumatic brain injury. Note: Proportion of respondents reporting exposure to major neurological conditions during residency or fellowship. Neurological pathologies covered in training.

Figure 1 summarizes the clinical pathologies covered during the participants' training. As shown, stroke and SAH were the most commonly encountered pathologies, with 95% exposure rates, followed by TBI (92%) and SE (88%). Other conditions, such as acute meningitis and spinal cord trauma, were less frequently observed, at 69% and 60% respectively. MG had the lowest exposure rate, at 59%.

### Self-reported clinical autonomy

Participants were asked in which clinical scenarios they felt capable of managing patients independently, without needing additional input from a colleague within the same specialty. This aimed to estimate the perceived impact of their NCC training on practical autonomy and clinical decision-making.

The conditions associated with the highest rates of self-reported autonomy were AIS (89%), brain death (84%), SE (82%), and CNS infections such as meningitis or encephalitis (78%). These conditions often form part of the core neurology training, which may explain the higher confidence levels.

**Figure 2:**
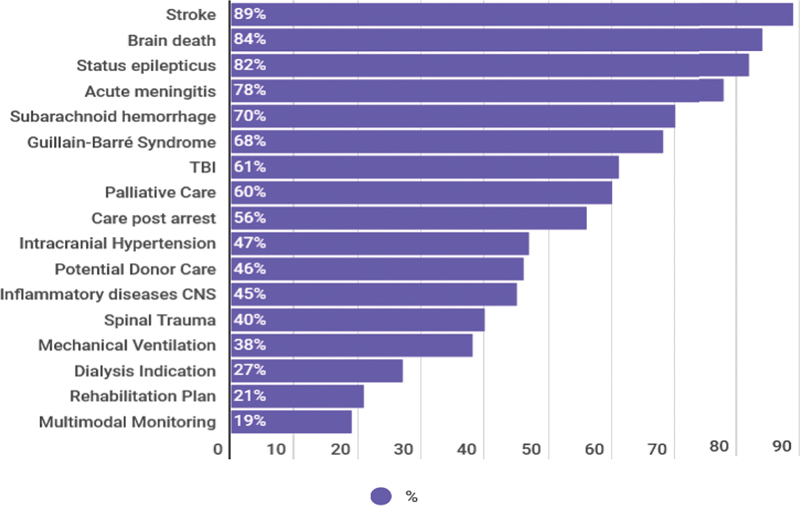
Abbreviations: TBI, traumatic brain injury; CNS, central nervous system. Note: Proportion of respondents who reported confidence to manage common neurocritical conditions without supervision, including stroke, subarachnoid hemorrhage, traumatic brain injury, intracranial hypertension, postcardiac arrest care, and spinal cord trauma. Clinical conditions professionals feel confident managing independently.

Conversely, autonomy was considerably lower for other prevalent and high-complexity pathologies. Only 70% felt confident managing SAH, and 61% for TBI, despite both being key neurocritical conditions. Guillain-Barré syndrome elicited similar autonomy rates (68%), possibly reflecting its higher visibility in tertiary centers. Neurological conditions in which participants felt confident they could act autonomously are presented in Figure 2 .

The lowest levels of autonomy were reported for postcardiac arrest care (56%), refractory intracranial hypertension (47%), potential organ donor care (46%), central nervous system (CNS) inflammatory diseases (45%), and spinal cord injury (40%). These figures suggest critical gaps in hands-on training for common ICU conditions with neurological impact.

Furthermore, when asked about general critical care practices, such as ventilator management, vasoactive drug titration, sedative weaning, end-of-life care, and rehabilitation planning, fewer than 70% of respondents felt confident acting independently. These findings reinforce the notion that most participants received limited exposure to comprehensive, integrated neurocritical care, particularly in aspects that overlap with general intensive care medicine.

## DISCUSSION

The current national Brazilian survey on NCC education during medical residency reveals concerning findings regarding both the prevalence and quality of training. Despite growing interest in the field, NCC remains largely absent from the formal curricula of neurology and intensive care medicine in Brazil. Training is delivered informally and heterogeneously, with limited institutional standardization. Our findings suggest that NCC education in Brazil remains a work in progress, with clear opportunities for strengthening both its institutional structure and curricular presence.

Although most responses were from professionals trained in large academic centers, particularly in the Southeast region, significant deficits were still identified. This likely overestimates the real national situation, as training opportunities may be even more restricted in underserved areas. The survey also showed that a considerable portion of residency rotations occurred in external institutions, suggesting that access to NCC education remains structurally limited to high-resource centers.

In contrast to the Brazilian model, NCC in countries such as the United States and parts of Europe is a formally recognized subspecialty. 13 Structured fellowships, standardized curricula, accreditation boards, and board certification mechanisms have contributed to high levels of professional autonomy. For instance, a US-based survey 17 found that over 90% of NCC graduates reported the ability to independently interpret systemic and cerebral hemodynamic data, perform neurovascular ultrasound, manage airways, and conduct a various of bedside procedures. In the present study, fewer than 85% of participants reported training in even basic procedures, and fewer than 30% had hands-on experience with advanced neuromonitoring tools such as EEG, ICP, or TCD.

This discrepancy may be related to a combination of factors, such as the tendency for procedural care to be performed primarily by senior staff, the emphasis on theoretical over practical teaching in many programs, and the limited availability of multimodal neurological monitoring in most hospitals. Moreover, even when participants were exposed to procedures or clinical domains, many reported not feeling confident performing them independently. This dissociation between exposure and autonomy was particularly evident in the management of SAH, intracranial hypertension, and postcardiac arrest care. Similarly, basic intensive care domains such as ventilator management, sedation, or palliative care showed autonomy rates below 70%, indicating insufficient practical depth, limited supervision continuity, and possibly a lack of structured progression toward clinical autonomy during training.

Another point of concern is the limited representativeness of the sample. Although the response rate was robust and included participants from multiple regions, the majority were neurologists, and respondents were primarily linked to large urban academic centers. This introduces a response bias; potentially inflating training rates and masking more severe deficits present in peripheral services. Moreover, the survey was distributed during academic conferences and professional forums, which may have been further selected for more engaged or better-trained professionals. These limitations should be taken into account when interpreting the findings.

Despite these challenges, the survey also revealed promising opportunities. Over half of the respondents expressed interest in pursuing NCC as a dedicated specialization. This enthusiasm reflects a potential base for developing structured training pathways. The implementation of accredited fellowship programs, the creation of a national core curriculum, and advocacy for NCC recognition by professional societies could promote access, visibility, and academic development.

Low-cost strategies may also offer substantial improvements. Structured clinical rotations in high-volume centers, faculty development programs, protocol-based teaching, and high-fidelity simulation training could bridge part of the current gap. Simulation has shown efficacy in improving both procedural skills and critical decision-making, especially for residents without continuous access to complex cases. 25 26

Looking forward, the advancement of NCC education in Brazil will require coordinated strategies to expand access, enhance training quality, and address regional disparities. Rather than awaiting formal subspecialty recognition, efforts could begin with structured pathways such as regional rotations, faculty development, and protocol-based teaching. Establishing hubs of excellence and fostering partnerships among residency programs, academic institutions, and public health agencies may provide a scalable and sustainable model. Active engagement from educational and regulatory bodies will be key to integrating NCC principles into existing curricula, ultimately strengthening the clinical preparedness of future neurologists and intensivists and improving care for critically ill neurological patients nationwide.

The present study represents the first national-level effort to assess the current state of neurocritical care education across multiple specialties in Brazil, offering valuable insights from a diverse sample of physicians trained in a wide range of institutions. The survey instrument was rigorously developed, piloted, and adapted from international standards, ensuring content validity and contextual relevance. Given the exploratory nature of the study and the sample size below the calculated threshold, we opted for a purely descriptive statistical approach. This choice aimed to provide a transparent and faithful representation of the data without inferring associations that could be statistically underpowered or misleading.

However, several limitations must be acknowledged. First, the voluntary and convenience-based nature of the survey may introduce response bias, as individuals more engaged in academic and NCC activities may have been more likely to participate. Second, although participation encompassed 14 Brazilian states, most respondents were concentrated in the more developed Southern and Southeastern regions. This geographic imbalance limits the generalizability of the findings to the national context, as training opportunities and infrastructure are likely to be even more restricted in less-resourced areas.

Furthermore, the final sample of 208 valid responses represents approximately 58% of the minimum target calculated a priori, which also constrains the precision of subgroup analyses and precludes robust inferential testing. Despite this limitation, the consistency of trends across specialties and regions supports the descriptive validity of the results.

Additionally, although the study captured self-reported autonomy and procedural exposure, it did not include objective assessments of competency or training outcomes.

Despite these limitations, the findings provide a foundational understanding of the current landscape and highlight urgent areas for policy intervention and curricular development. Moving forward, longitudinal studies and formal needs assessments are warranted to guide the implementation of national training standards and certification frameworks, as well as to evaluate the impact of educational reforms on patient outcomes.

In conclusion, NCC education in Brazil remains fragmented and heterogeneously implemented. Structural, curricular, and institutional limitations contribute to insufficient practical training and wide variability in clinical autonomy. Nevertheless, growing professional interest, combined with a clear recognition of the educational gap, suggests strong potential for development. Investing in structured training pathways, accessible faculty development, and national-level educational planning will be crucial to supporting equitable access to high-quality NCC education nationwide.
